# Staff Perspectives Toward Challenges in a Newly Established Cancer Center in Tanzania: A Qualitative Study

**DOI:** 10.1200/JGO.18.00246

**Published:** 2019-04-03

**Authors:** Zainab Alwash, Oliver Henke, Furaha Serventi, Eva Johanna Kantelhardt

**Affiliations:** ^1^ Institute for Tropical Medicine and International Health, Charité Universitätsmedizin Berlin, Berlin, Germany; ^2^ Cancer Care Centre, Kilimanjaro Christian Medical Centre, Moshi, Tanzania; ^3^ Institut für Med. Epidemiologie, Biometrie u. Informatik Medizinische Fakultät, Martin-Luther-Universität Halle-Wittenberg, Clinical University of Halle (Saale), Halle (Saale), Germany

## Abstract

**PURPOSE:**

Cancer is a growing public health concern in low-income countries (LICs). From 14 million new patient cases identified worldwide each year, 8 million are diagnosed in LICs. The fatality rate is 75% in LICs compared with 46% in high-income countries. Causes are low literacy levels, lack of awareness and knowledge about cancer, and limited education of health care professionals that leads to late detection and diagnosis. In Tanzania, cancer incidence will double to 60,000 in 2030. The referral hospital of Northern Tanzania established a new cancer unit in December 2016 to meet these needs. However, there is limited knowledge about perceptions of health care professionals toward cancer care in LICs. This study aims to understand attitudes and perspectives of those professionals and the treatment-related challenges in a newly established center to assist future efforts in this field.

**METHODS:**

A qualitative method approach using in-depth interviews was chosen to achieve inductive conceptualization. Analysis of data was performed according to qualitative content analysis.

**RESULTS:**

Eleven interviews were conducted. Five main categories were found: training and education of staff, availability of financial support, challenges in management, interests in future developments, and job satisfaction. Subcategories elaborated in more detail within the main categories.

**CONCLUSION:**

Limitations in staffing, training, and education were major concerns. The importance of sustainable funding and the needed cooperation of the government with international aid were identified as key points. The involvement of different stakeholders requires guidance by health care management. Health care professionals expressed their satisfaction with the possibilities of treating cancer and the rewarding feedback from patients. Misconceptions and poor knowledge by patients were mentioned as reasons for delayed health-seeking behavior. Screening and awareness programs were seen as useful interventions.

## INTRODUCTION

Although, in the past, cancer was almost exclusively an issue for developed countries, the American Cancer Society states that it is becoming more of a global health concern.^1^ Cancer incidence in low-income countries (LICs) is increasing because of multiple factors, such as Westernization, changes in lifestyle, increased life expectancy, and advances in diagnostic and detection practices.^2^ Half of the global cancer incidence (8 million) in 2012 was diagnosed in LICs according to the International Agency for Research on Cancer.^3^ For Tanzania, cancer incidence is expected to nearly double in only 15 years, from 37,000 new patient cases in 2015 to more than 61,000 in 2030.^3^

Tanzania is one of the poorest countries in the world. A report by the Tanzanian National Bureau of Statistics in 2013 indicates that 28.2% of the population lives below the basic needs poverty line; 68% live on less than 1.25 US dollars a day, according to the World Bank.^4,5^ Cancer aggravates the cycle of poverty, especially in countries with limited resources and weakened health systems.^6^ Poverty is a notable barrier for seeking early medical attention.

Another important barrier to seeking early medical attention is poor literacy. Lack of awareness and knowledge about cancer is highlighted by the fact that only a minority of the population in Tanzania considers cancer a major health problem.^6,7^

Tanzania has a total population of almost 55 million^5^ but has only 0.31 doctors per 10,000 people to provide medical services.^8^ Until recently, Tanzania was home to only two specialized cancer hospitals: Ocean Road Cancer Institute (ORCI) in cooperation with Muhimbili National Referral Hospital in Dar Es Salaam and Bugando Medical Centre in Mwanza. Both hospitals face staff and equipment shortage,^9^ which makes it more challenging to provide quality health services to a large number of patients.

Regarding training and education, two postgraduate Masters programs at Muhimbili National Referral Hospital are available: Master of Medicine in Clinical Oncology and Master of Medicine in Hematology and Blood Transfusions. Postgraduate hematology-oncology education for nurses is not available in Tanzania, but efforts have been made to introduce short educational courses.^10^

In the past decade, the Tanzanian government has recognized the need for cancer treatment and accordingly started funding ORCI to support early detection programs and to provide services to patients who need chemotherapy and radiation treatment. However, the number of patients is exceeding the capacity of the hospital.^7^

With the initiative to address the needs of patients with cancer, Kilimanjaro Christian Medical Centre (KCMC), the tertiary hospital of the Northern Zone of Tanzania, established a new cancer care center (CCC) in December 2016.

Establishment of the CCC is planned in three phases. The first phase, which started in December 2016, provided an outpatient clinic and chemotherapy infusion unit. Although many patients are also treated as inpatients in their respective departments, these inpatients are seen by the specialists on a consultation basis. The second phase, which is starting in 2019, will be the construction of an oncology inpatient ward and a patient hostel, which will be instrumental for patients who do not need inpatient care but need to travel long distances for treatment. The final, third phase, which is planned for 2021, will be a radiation unit.

During the first year of operation, two specialized physicians, four nurses with oncologic training, one specialized palliative care nurse, two pharmacists, one administrative staff, and one public health officer were employed at the CCC. Approximately 900 patients attended the service at CCC in more than 3,000 appointments, and 400 patients received chemotherapy in 2017.

Funding of the CCC is a joint effort lead by the US-based Foundation for Cancer Care in Tanzania, the Tanzanian Government, and the Evangelical Lutheran Church of Tanzania with their partnering organizations in Germany.

Cancer treatment with all its implications has not been widely studied in Sub-Saharan Africa. Cancer treatment is a new medical service provided by the CCC, so this study aims to document and analyze the attitudes and perspectives of health care staff toward chemotherapy and the challenges they face on a daily basis at their workplace. This understanding will help explore the service-related spectrum of challenges that occur in LIC settings.

## METHODS

### Methodologic Approach and Study Design

The research field is widely unknown, so we chose the grounded theory approach to “elicit each participant's interpretation of his or her experience.”^11^ This approach assures an understanding of the participants’ viewpoints and their own concepts of work in cancer care. By using in-depth interviews as a qualitative and comprehensive approach, an inductive conceptualization of the topic can be achieved.

### Sampling and Participants

All professional staff members working for the new CCC when the study was conducted were interviewed. The characteristics of the participants are listed in Table 1.

**TABLE 1:**
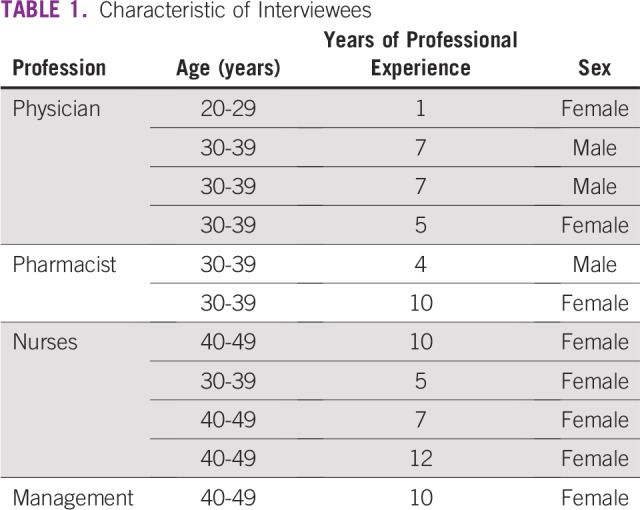
Characteristic of Interviewees

### Data Collection and Analysis

After extensive narratives of the participants were obtained through open-ended questions, semistructured interviews followed to focus on specific topics: the experience of each participant about handling chemotherapy and working at the CCC, the challenges he or she faced on a daily basis, and possible suggestions for improvement.^12^ The interview guideline was based on literature review and discussions made with key persons in and outside KCMC before data collection. The professional background of the interviewees was taken into consideration during the guideline design and during the interviews.

Analysis of data was performed according to qualitative content analysis and followed the steps of inductive theme formation, context analysis, and structuring.^13^ Analysis was conducted by two researchers independently to assure validity.

The study was approved by the board of the Ethic Committee of Kilimanjaro Christian Medical College. The study also followed guidelines of Good Research Practices according to the Declaration of Helsinki of the World Medical Association.

## RESULTS

In total, 11 interviews were conducted between March and May 2017. Interviews were conducted by the first author and lasted between 20 and 60 minutes.

As a result of the interview analysis, five main themes were identified: training and education of staff, availability of financial support, challenges in management, interests in future development, and job satisfaction; corresponding subthemes were explored (Table 2). Corresponding quotations are displayed in Table 3.

**TABLE 2:**
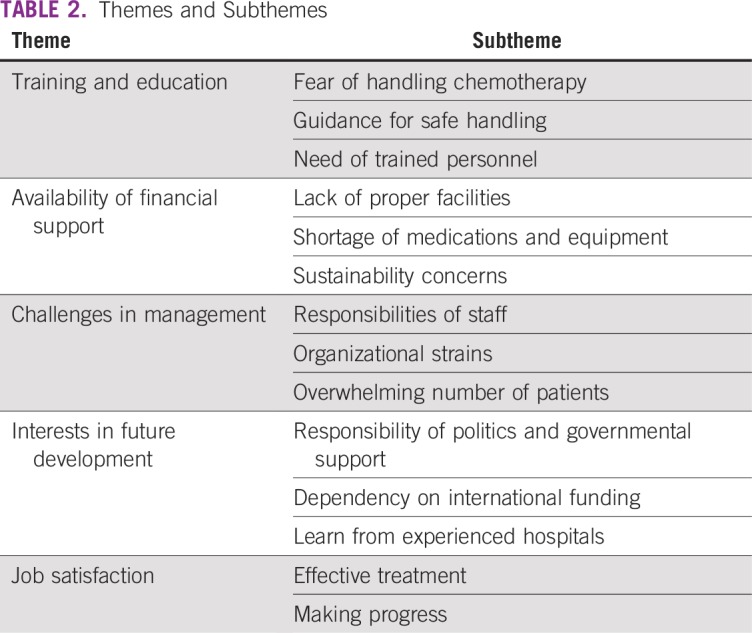
Themes and Subthemes

**TABLE 3:**
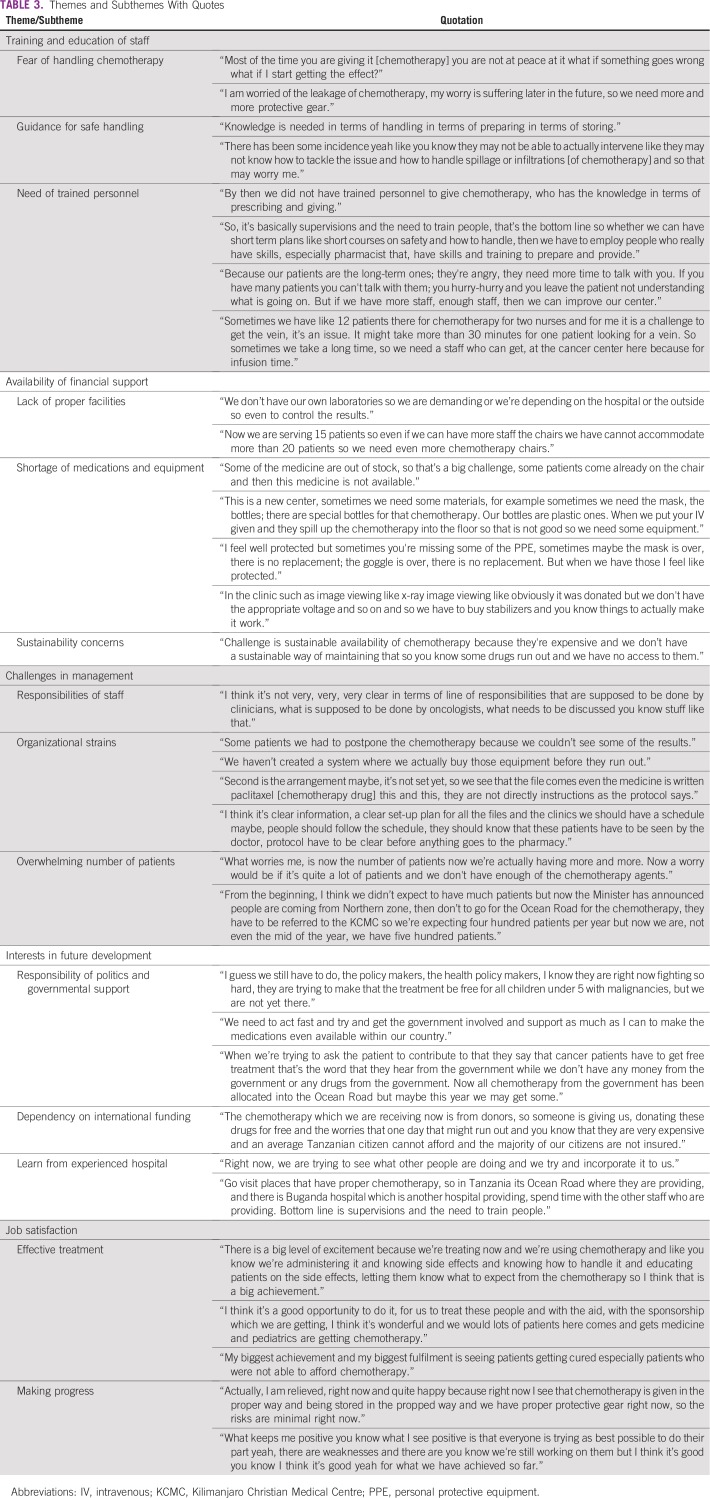
Themes and Subthemes With Quotes

### Training and Education of Staff

This main theme contains all assertions about professional training of the interviewees themselves or of the CCC staff in general. In addition, the theme contains related issues connected to professional education.

#### Fear of handling chemotherapy.

One of the most prominent findings, mentioned by every participant, was fear of handling chemotherapy. Health care staff expressed distress about handling chemotherapy: they feel concerned about a high risk of suffering toxic effects of the medication in the future as a result of exposure, especially when they prepare the chemotherapy without using proper protective equipment. This issue was not asked about by the interviewer in particular; however, it was mentioned in response to the open-ended question.

#### Guidance for safe handling.

Because this was a new field for the staff, they mentioned a critical need for guidance about how to handle chemotherapy safely and properly and how to store it.

#### Need of trained personnel.

The interviewees shared a common view that the CCC was understaffed, especially with the increasing number of patients. In addition, the staff expressed their need for more training.

Conversely, some nurses mentioned their worries about not giving patients optimal counseling because of limited time capacities. They also expressed that adequate training and increased staff members would lead to a better outcome.

Some of the nurses expressed their difficulties in venipuncture, especially when under time pressure. Sometimes venipuncture required more time for each patient, so it added more stress about the understaffing issue.

### Availability of Financial Support

Financial difficulties and limited resources were mentioned by the CCC staff in different ways. They expressed the need for more financial support to improve health care facilities, to increase the availability of medications and equipment, and to provide assistance to patients with cancer.

#### Lack of proper facilities.

CCC staff noted their need for improved facilities that would offer better infrastructure, such as a laboratory for the cancer center with standardized testing procedures and more equipped chemotherapy rooms.

#### Shortage of medications and equipment.

Some of the health care staff stated their concerns about the availability of medications and of necessary equipment for safe-handling procedures. Although donations can be helpful for the newly established CCC, especially because it operates in an LIC setting, some donations were not fit for the purpose and not suitable for the setting.

#### Sustainability concerns.

Apprehensions were expressed about the sustainability of the CCC service, because it is based mainly on international funding.

### Challenges in Management

The staff faced various operational issues that were hindering efficiency in the work place. In particular, the dynamics of a newly established cancer treatment facility, with many different stakeholders involved, would lead to unforeseeable hurdles. The following subthemes were identified:

#### Responsibilities of staff.

Staff noted that division of labor and definition of tasks were not clearly enough defined between different specialties, which caused duplicate or unattended responsibilities.

#### Organizational strains.

The other management issue mentioned was lack of organizational arrangements on a day-to-day basis at the CCC, and its effects were noticed in many aspects, such as delays in the patient test results. A physician mentioned that CCC was still going through phases of out-of-stock equipment because of the lack of organizational skills to audit the stock and order equipment timely. Use of more standardized measures was mentioned as a way to enhance efficiency for the daily work routine.

#### Overwhelming number of patients.

Unprecedented, overwhelming numbers of patients for diagnosis and treatment created more challenges in the management of the center, as mentioned by many interviewers.

### Interests in Future Development

The health care providers expressed their huge interest in the development of the CCC, and they identified some of the intertwined factors that played a paramount role in the development process:

#### Responsibility of politics and governmental support.

The care providers mentioned the importance of the local government’s role in providing future support to the CCC. This support could be manifested in policy making, such as financial support to the Tanzanian citizens diagnosed with cancer.

Another way in which the government could play a role is by assisting in the provision of medications and by making medicine available on a local level. One of the participants mentioned that the financial support was directed to the government-run ORCI. However, as per the government plan, the target was to decentralize cancer services to other regions in the country. This process, however, has not yet reached KCMC, and the delay was creating financial strains.

#### Dependency on international funding.

The health care staff expressed their current worry about their dependency on international donors to provide the needed medications and equipment. This dependency raises concerns about sustainability and continuity of the provision of services in the future.

#### Lessons from experienced hospital.

Some of the health care providers proposed learning from other hospitals that have experience in this field and a similar low-resource setting, like ORCI or the Bugando Medical Centre. This suggestion could be helpful because of the common context that these centers share.

### Job Satisfaction

Besides the challenges and obstacles in the delivery of chemotherapy, the interviewees expressed their satisfaction about working at the CCC.

#### Effective treatment.

The possibility of having an effective treatment of cancer, which was lacking, was an exciting experience for the health care professionals (HCPs). The joy of getting a rewarding feedback by curing patients or at least by reducing their burden of symptoms also was mentioned, especially by the nurses.

#### Making progress.

Regardless of the problems mentioned, the staff of the new established CCC appreciated the progress in their field of work.

### Differences Among Groups of Interviewees

Generally, the answers of the HCPs were consistent throughout all professional groups. With regard to the fear of handling chemotherapy, responses depended on previous experience and level of exposure to chemotherapy. Those HCPs involved in handling, reconstitution, or administration of chemotherapy expressed more concerns. Female professionals were especially concerned about the influence of handling chemotherapy on their reproductive health.

## DISCUSSION

This study explored the attitudes and perspectives of health care staff toward cancer care in a newly established treatment facility in a low-resource setting.

One theme mentioned by all participants was the strong need for more oncology training. This finding resembles results of previous research. In a Knowledge, Attitudes, and Practices study conducted with medical staff in Uganda, less than 40% were aware of the risk factors for cervical cancer.^14^ Rick et al^15^ displayed that the baseline knowledge among HCPs about cancer was limited in an urban Tanzanian setting. Additional studies have shown that poor baseline understanding of cancer and chemotherapy exists among medical staff in Tanzania and concluded that there was a need for continuous medical education and training.^15-17^ The same findings were highlighted by Makani et al^18^ about knowledge in hematology among Tanzanian HCPs.

In this study, many participants mentioned that sufficient oncology training was lacking before they started working at CCC. Thereby, the nurses in particular played an important role to provide care and deliver services to patients through monitoring of physical conditions and administration of chemotherapy. In addition, they helped counsel patients and families about medical conditions and the expected adverse effects of the treatment.

Despite partial funding by international donors, the insufficient level of financial support at CCC was another main concern. That manifests itself in two dimensions: the lack of proper facilities and shortages of medication and equipment, and the concern about future sustainability of these international funds and so the continuity of the CCC itself.

The financial concern is closely interlinked with the above-mentioned fear of handling chemotherapy: When inadequately trained staff and a lack of standard operating procedures are coupled with equipment shortages (in particular, protective gear), HCPs lack confidence to perform daily tasks, and their fear is reinforced.^19^

However, the interviewees expressed satisfaction with the achievements so far, especially that effective treatment options were available for patients with cancer. Furthermore, rewarding feedback from the patients was another positive aspect for the HCP. These findings are consistent with a study from Kamisli et al^20^ among Turkish oncology nurses, in which nurses indeed expressed their exhaustion but also their increased levels of empathy, patience, awareness about priorities in life and job satisfaction. Despite an excessive workload, Australian oncology nurses showed high levels of personal satisfaction and personal accomplishments in a survey among 234 nurses.^21^ That job satisfaction among this group can be enhanced by a good physician-nurse relation, the freedom to make patient-care decisions, and appropriate staffing was stated in a Canadian survey.^22^

Health care management was another concern for the HCPs. This concern manifested as difficulties in efficient provision of services, such as having test results prepared before the next chemotherapy cycle, having an electronic inventory system for medications to have necessary stock always available, and not having standardized treatment protocols.

Management and leadership are vital for good health governance. As a health governance report by the United States Agency for International Development stated, it is important to have governance in addition to operational capacity when delivering health services.^23^ Organizational strains, lack of recognition and support from management, and unrealistic expectations were also associated with lower job satisfaction and higher burn-out rates among New Zealand oncology HCPs.^24^

Limited management capacity was negatively affected by the unprecedented number of patients visiting the center. When the study was conducted, only four nurses and one full-time pharmacist were assigned to CCC to provide services for approximately 180 chemotherapies monthly. As of 2018, after the increased public awareness of cancer that resulted from media coverage of the services provided, CCC expected to receive approximately 600 additional patients annually, according to the executive director of KCMC.^25,26^ This increase could create more strains on the availability of medications and equipment and also may affect the quality of services.

The study results were used to shape the functionality of the center. Standard operating procedures for chemotherapies have been implemented, and managerial issues were resolved by implementing the position of a clinic administrator who oversaw funding, procurement, and patient flow. A training about safe handling of chemotherapy conducted by the German Institute for Medical Mission was extended to respond to the fear of handling. Continuing medical education for the staff was implemented as internal and external trainings in regular intervals. Currently, staff numbers have increased and the government has contributed to medication on a large scale.

Although all interviewees received their professional education in the English language, a limitation could be loss of information by not presenting information in Swahili. The study was conducted shortly after the opening phase of the CCC. Therefore, the results must be interpreted with this background. Nonetheless, this timing is also a strength, because it provided insight about the problems during the implementation phase and could serve as a guidance for similar centers.

As cancer burden increases continuously, increasing numbers of patients in the few available cancer treatment facilities in Tanzania are expected. Adequate numbers of qualified HCPs must be trained and available from the early stages of new facilities. Provision of standard operating procedures for cancer treatment could help streamline daily work tasks. The NCCN guidelines for Sub-Saharan Africa^27^—as well as the national guidelines of Tanzania, which are expected to be released in 2019—could be instrumental. Because cancer care in an LIC is costly, coordination with health insurances, donors, non-governmental organizations, pharmaceutical companies, and other stakeholders is an important management task that must be addressed by health care management professionals.

Collaboration with existing cancer treatment facilities in an LIC can provide expertise and synergy for new centers. To create resilience among oncology nurses, an interventional study from Poulsen et al^28^ showed that a 1-day interventional workshop has the potential to enhance resilience, prevent burn-out, and increase satisfaction with current self-care.
